# Routineness of Social Interactions Is Associated With Higher Affective Well-Being in Older Adults

**DOI:** 10.1093/geronb/gbae057

**Published:** 2024-04-10

**Authors:** Minxia Luo, Kristina Yordanova, Birthe Macdonald, Gizem Hülür

**Affiliations:** Department of Psychology, University of Zurich, Zurich, Switzerland; Healthy Longevity Center, University of Zurich, Zurich, Switzerland; Data Science Institute, University of Greifswald, Greifswald, Germany; Department of Psychology, University of Zurich, Zurich, Switzerland; University Research Priority Program “Dynamics of Healthy Aging,” University of Zurich, Zurich, Switzerland; Department of Psychology, University of Bonn, Bonn, Germany; (Psychological Sciences Section)

**Keywords:** Activity engagement, Cognition, Event-contingent experience sampling, Positive and negative affect, Recurrence quantification analysis

## Abstract

**Objectives:**

Some research conceptualizes routineness of daily life as an indicator of cognitive vulnerability that would lead to lower well-being in older age, whereas other research expects routineness to give rise to more meaning and stability in life and thus to higher well-being. Further research is needed to understand routineness in older adults in relation to cognitive abilities and well-being. This study examined routineness of social interactions.

**Methods:**

We examined data from an event-contingent experience sampling study with 103 Swiss community-dwelling older adults (aged 65 to 84 years). Participants completed in-lab cognitive assessments (reasoning, episodic memory, speed, and vocabulary) and reported their well-being (positive affect, negative affect, and life satisfaction). For more than 21 days, participants reported the time and context of their social interactions (including modality, partner type, and location). Routineness of social interactions was defined as social interactions that occurred at the same time of day over the study period. It was calculated using recurrence quantification analysis.

**Results:**

Linear regressions showed that higher routineness of social interaction in general, of social interaction through the same modality, and of social interaction with the same partner type were associated with higher positive affect. Higher routineness of social interaction in general was associated with lower negative affect. Routineness of social interactions was not associated with life satisfaction or cognitive abilities.

**Discussion:**

A routine social life may increase older adults’ affective well-being. Results are discussed in the context of activity engagement and time use in older age.

One of the major aims of gerontology research is to provide older adults with advice on what to do in their free time, especially after retirement, so as to maximize their health and well-being (Havighurst, 1963; Horgas et al., 1998; Oerlemans et al., 2011). Thus, it is important to study older adults’ daily time use and understand the role of time and energy allocation in relation to health and well-being (Luo, Macdonald, et al., 2022; Möwisch et al., 2022). As one way to quantify patterns of daily time use, research on routineness of activity engagement has long existed in the aging literature (Zisberg et al., 2007, 2009) and has received renewed interest (Mohideen & Heintzelman, 2023; Tighe et al., 2015). Routines refer to relatively fixed temporal patterns of an individual’s activities (Clark, 2000).

Social interaction is an important daily activity to spend time on at any age, especially in older age (Charles et al., 2021; Stine-Morrow & Manavbasi, 2022). In particular, the socioemotional selectivity theory proposes that, when older adults have a limited future time perspective, they would prioritize the engagement in social interactions that are emotionally meaningful (Carstensen, 1995; Lang, 2001). Nurturing relationships necessitates shared routines among social ties (Fiese et al., 2002). Routineness of social interactions has not been examined in older adults. This study aims at filling this gap by examining routineness of social interactions in relation to cognition and well-being in community-dwelling older adults’ daily life.

## Cognitive Ability and Routineness

Routinized behaviors were viewed as a way to reduce complexity of information to be processed and, thus, a reflection of inflexibility or rigidity in the aging process (Tournier et al., 2012). Moreover, preference for routineness was understood as an indication of cognitive vulnerability in aging research (Reich & Zautra, 1991; Zisberg et al., 2009). For instance, higher preference for routines was more likely to be found in community-dwelling older adults with more cognitive complaints and more cognitive decline (based on the Mini-Mental State Examination; Bergua et al., 2006). Higher preference for routines was more likely to be found in older adults (who were students at a senior university) with worse performance in tasks of divided attention, which was interpreted as a lack of cognitive flexibility (Tournier et al., 2012). Additionally, higher preference for routines was found in older adults (living at home) with lower functional status in completing tasks of daily living, such as shopping and managing finances (Bergua et al., 2013; Zisberg et al., 2009). Thus, higher routineness can be expected to be associated with lower cognitive abilities in older age.

According to life-span developmental perspectives, individuals play an agentic self-regulatory role in their own development (Baltes & Carstensen, 1996). In the face of aging, older adults may use adaptive strategies to bridge between needs, available resources, and contextual constraints (Baltes et al., 1999). Cognitive ability is one form of available resources that are necessary for everyday functioning (Baltes & Lang, 1997). In terms of routineness of social interactions, the engagement in social interactions requires energy (Hall & Davis, 2017; Luo, Pauly, et al., 2022). Social interactions that occur regularly could reduce older adults’ efforts in coordinating their time schedules. Thus, developing routines might be an adaptive strategy for older adults to maintain their social relationships, in face of cognitive decline in aging.

## Routineness and Well-Being

In line with the assumption of declining resources in older age, older adults might be forced to reduce complexity of their daily life and to replace it with routines and predictable activities (Bergua et al., 2013). This lifestyle may be filled with sameness and repetition and thus routineness could lead to lower well-being. For example, higher preference for routines was associated with higher trait anxiety and depression in older adults living independently and in residential facilities (Bergua et al., 2006; Bouisson, 2002). Moreover, repetitive activities and contexts were associated with lower happiness in older adults living at home and in retirement homes (Bouisson & Swendsen, 2003).

However, recent research on meaning of life proposes that enactment of routineness gives rise to sense of life meaning through connecting a person to a larger context, which offers a structured framework for individuals to interpret their experience (King & Hicks, 2021). As quoted in prior literature (Heintzelman & King, 2019; Zisberg et al., 2007), “Human beings derive meaning and maintain well-being through the organization of time” (Meyer, 1922, p. 6). In line with this view, a study showed that routines were associated with higher sense of meaning in life in young adults (Heintzelman & King, 2019). A recent study with young adults further showed that the positive association between routines and meaning in life existed, independent of the content (i.e., relationship closeness, goals, religious, provinciality) and contexts (difficult times, e.g., COVID-19 pandemic) of the routines (Mohideen & Heintzelman, 2023).

Relatedly, according to the social zeitgeber theory in understanding life events (e.g., spouse’s death) and mental disorders, “social Zeitgebers” refer to “personal relationships, social demands or tasks that serve to entrain biological rhythms” (Ehlers et al., 1988, p. 948). In other words, routineness of social interactions is a reflection of a stable life without unpleasant disruption (e.g., social interactions being disrupted) that leads to irregular biological rhythms, such as sleep–wake cycles (Ehlers et al., 1988; Grandin et al., 2006). Research has shown that higher routineness in daily life is more likely to be observed in healthy older adults, in comparison to stroke patients (Campos et al., 2008) or older patients with major depressive disorder (Lieverse et al., 2013). Similarly, higher routineness was related to higher levels of life satisfaction and lower levels of depression, anxiety, and stress in adults with average ages at midlife (Margraf et al., 2016). Accordingly, higher routineness could lead to higher well-being.

Research on associations between routineness and well-being is inconsistent. Whereas some research sees routineness bringing rigidity to life and is associated with lower well-being, other research sees routineness as a way to achieve higher well-being. Further research is needed to understand routineness in older adults’ daily life in relation to well-being.

## Examining Routineness in Daily Life

There have been different approaches to quantify routineness of activity engagement in daily life. For example, a daily diary-like activity sheet (i.e., Social Rhythm Metric) was developed to collect information of a selected list of activities and with whom each activity was carried out, such as time out of bed and time of first contact with another person (Monk et al., 1991, 1994). In this method, routineness was defined by counting the number of events occurring within 45 min of the habitual time of day (defined as occurrence more than three times per week; Monk et al., 1991). Another study invited older adults to describe their present activities and contexts four times per day for more than 4 days (Bouisson & Swendsen, 2003). Researchers manually coded different categories of activities (e.g., shopping, leisure) and contexts (e.g., with friends, with strangers) and quantified routine as repeated activities or contexts observed within “the same time period” across days, although duration of the period was unspecified. Another study asked university students to report multiple times per day the degree of routineness of the current activity by endorsing statements such as “the activity I’m doing right now is a part of a routine I have” (Heintzelman & King, 2019, p. 692).

According to a systematic review on the concept of routine, routines consist of repetitive behavioral patterns that are organized within the axes of time and space and occur in social and physical–spatial contexts (Zisberg et al., 2007). Similarly, the above-mentioned studies defined routineness as activities, contexts, or activities plus contexts that occurred at the same time of day (Bouisson & Swendsen, 2003; Monk et al., 1991). Thus, routineness of an activity could be quantified by the timing of an activity itself or in combination with its contexts. However, the data collection methods of the above studies were suboptimal. Specifically, the method of Social Rhythm Metric (or a daily diary method) largely relied on retrospective memory, which is known to be subject to retrospective bias. Additionally, the signal-contingent experience sampling design (data collection at predefined time points) may have overlooked events that occurred between measurement points and led to inaccurate understanding of routineness.

## The Current Study

This study examined routineness of social interactions in a sample of community-dwelling older adults. Social interaction is one of the commonly occurring activities in older adults’ daily life and is important for their well-being (Möwisch et al., 2022). Based on the life-span developmental perspective (Baltes & Carstensen, 1996), we viewed older adults as self-regulatory agents who make decisions regarding their organization of daily social interactions. First, we examined whether and how cognitive abilities (including reasoning, episodic memory, speed, and vocabulary) were associated with routineness of social interactions. As lower cognitive abilities might force older adults to establish routines, we expected that higher routineness would be associated with lower cognitive abilities. Second, we examined whether and how routineness of social interactions was associated with well-being (positive affect, negative affect, and life satisfaction). We expected that enactment of routineness could be associated with older adults’ well-being. Because existing theories and evidence are inconsistent, we did not formulate a directed hypothesis on the associations between routineness and well-being.

We examined data from a 21-day event-contingent experience sampling study on social interactions (Macdonald & Hülür, 2020; Hülür et al., 2023). The event-contingent experience sampling method asks participants to complete a prompt whenever they experience an event of interest and has been shown to be advantageous for accounting for the timing of social interactions (Himmelstein et al., 2019). That is, it minimizes retrospective recall bias introduced by a daily diary method (Margraf et al., 2016) and overcomes the limitation of a signal-contingent design, which possibly overlooks events occurring between measurement points (Bouisson & Swendsen, 2003).

**Figure 1.**
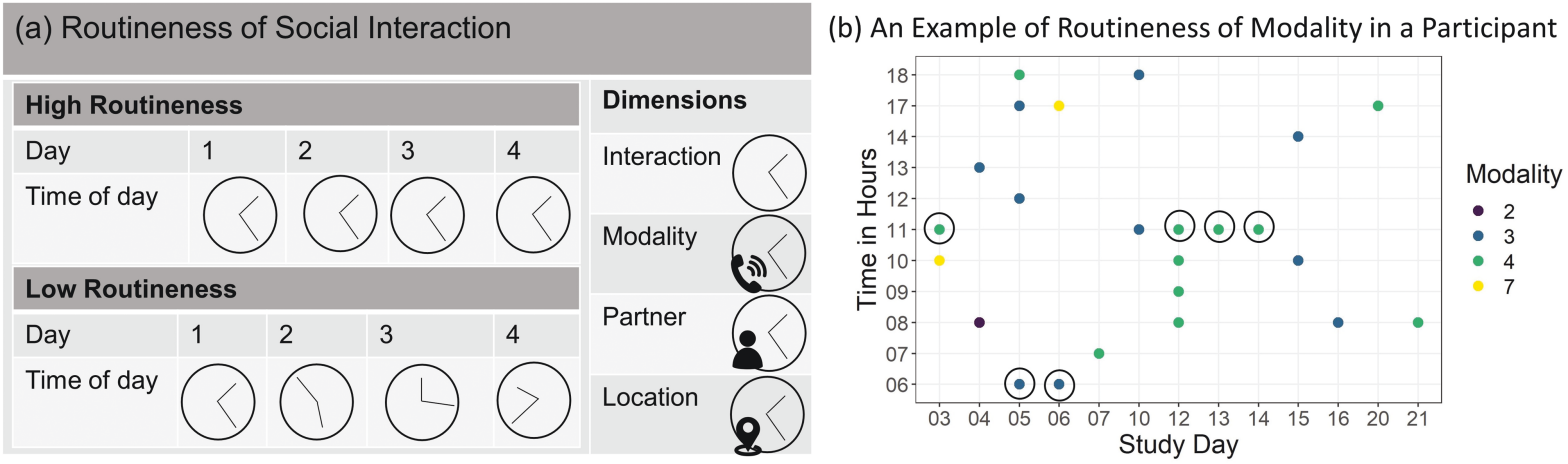
Operational definition of routineness of social interactions. Routineness was defined as the degree that predefined events happened at the same hour of day across different days. Routineness of social interactions was quantified by four different dimensions (panel A): social interactions in general, interaction modality, partner type, and location. Panel B shows a participant’s observations regarding interaction modality. X-axis displays the study day in which an interaction was observed. Y-axis is the time of the interaction in hours. Modality depicts the type of interaction (2: “Phone,” 3: “E-mail,” 4: “SMS,” 7: “Social media”). Recurrent interactions are depicted with a double circle.

Similar to prior research (Bouisson & Swendsen, 2003; Monk et al., 1991), we referred to routineness as the degree that the event of interest happened at the same time (i.e., hour) of day across different days. As seen in Figure 1 panel A, we defined the event of interest by the activity itself (i.e., social interaction in general) and by the activity plus the context, including communication modality (Macdonald et al., 2021), interaction partner, and location (Monk et al., 1991). That is, we examined whether social interaction in general, social interactions through the same modality, with the same interaction partner type, and at the same location occurred on the same hour across days.

## Method

### Participants

Participants were recruited via advertisements in local and national newspapers and through a database of participants hosted at the University of Zurich. Participants aged 65 years or older were recruited according to the following inclusion criteria: using digital devices to communicate, having sufficient hearing and vision, and being fluent in German. Participants were compensated with 150 Swiss Francs for their participation.

A total of 120 older adults from German-speaking regions of Switzerland participated in the study. We excluded six participants’ data because one did not complete the take-home questionnaire, one misunderstood the experience sampling protocol, two had missing data on their health conditions, and two were not retired. We further excluded 11 participants who had extreme scores in the variables of interest. Scores with a distance of larger than 1 *SD* away from the next neighboring score were excluded as outliers. The final sample included 103 participants.

## Study Design and Procedures

Participants took part in a baseline session where they received detailed instructions on the study protocol and a take-home questionnaire to collect their demographic information and psychological variables. Participants were given an iPhone 4S with the app “iDialogPad” (G. Mutz, Cologne, Germany) to complete a 21-day event-contingent experience sampling study. Participants were asked to report any spoken interactions (i.e., face-to-face, telephone, video chat) that lasted longer than 5 min and any text-based conversations (i.e., text message, e-mail, letter, social media). The 5-min cutoff was implemented based on earlier research on meaningful social interactions in daily life (Reis & Wheeler, 1991). Participants were instructed to report each social interaction as soon as possible after it happened. It was possible to report social interactions that took place up to 3 days ago with the questions “[d]id the conversation take place today?” (Yes/No); and if not, “when did the conversation take place?” (1 day ago/2 days ago/3 days ago). Based on the data from the 103 participants included in the current study, over 95% of interactions were reported on the same day. The average time gap between the time when a social interaction was reported and the time when the social interaction happened was 2.98 hr (*SD* = 3.35 hr). Finally, participants returned to the laboratory and completed a battery of cognitive assessments.

Data collection took place from April 2019 to November 2019. The study procedures were conducted according to the Declaration of Helsinki and were approved by the Ethics Committee of the Faculty of Arts and Social Sciences at the University of Zurich (Nr. 19.2.17). Written informed consent was obtained from all the participants.

## Measures

### Social interactions

Any conversation with the same person within the same context was defined as one social interaction, even if it occurred intermittently. It did not necessarily involve engaging in a lengthy discussion, but could involve exchanging a few words now and then. *Interaction time:* Participants reported the hour and minute of each social interaction, in the format of “hh:mm.” Interaction time was extracted as hours, ranging from 0 to 24. *Interaction modality:* For each social interaction, participants answered the following question: “How did the conversation take place?” They could choose one of the following options: (1) face-to-face (51%), (2) telephone (16%), (3) e-mail (12%), (4) text message (17%), (5) video chat (1%), (6) letter (1%), and (7) social media (2%). *Interaction partner type:* Participants reported “who was part of the conversation?” by choosing one of the following options: (1) known person (84%), (2) unknown person (10%), and (3) service provider (someone between a complete stranger and someone with a personal relationship, e.g., someone who provided support for health, transportation, repairs, or IT; 6%). *Interaction location:* Participants reported the place of the interaction by choosing either (1) private (69%) or (2) public place (31%).

### Cognition

The most important facets of adult intelligence were assessed, including reasoning, episodic memory, speed, and vocabulary (Schaie, 1994). *Reasoning* was assessed with five tests (*M* = 0.05, *SD* = 0.71, range = −1.45 to 1.99, McDonald’s omega = 0.76). In a numeric reasoning task, participants inserted operators in simple mathematical equations with plus and minus signs missing (Süß & Beauducel, 2015). In a task on recognizing rules, one of eight characters in each line contradicted an underlying rule. Participants recognized the rule and crossed out the wrong character (Sturm et al., 1993). In a computational reasoning task, participants solved mathematical text problems (Süß & Beauducel, 2015). In a route memory task, participants memorized the route between two places marked on a city map and subsequently drew the route on an unmarked map (Süß & Beauducel, 2015). In task on verbal analogies, participants completed word analogies (Süß & Beauducel, 2015).

*Episodic memory* was assessed with two tests (*M* = 0.05, *SD* = 0.79, range = −1.67 to 2.45) (see Author Note 1). In a word recall task, a list of words had to be memorized and reproduced in free order (Süß & Beauducel, 2015). In a numerical memory task, a list of pairs of three-digit numbers had to be learned. At retrieval, the first number of each pair was given in a different order, and the second number was to be recalled and written down (Süß & Beauducel, 2015).

*Speed* was assessed with three tests (*M* = 0.07, *SD* = 0.76, range = −2.50 to 2.14, McDonald’s omega = 0.67). The digit symbol test required participants to write down the corresponding symbol as fast as possible under each digit according to nine digit–symbol pairs followed by a list of digits (Wechsler, 1955). In an animal naming task, participants wrote down as many names of animals as quickly as possible (Tombaugh et al., 1999). In a word classification task, participants marked all words naming plants in a list of words (Süß & Beauducel, 2015).

*Vocabulary* was assessed with multiple choice tasks (*M* = 32.13, *SD* = 2.64, range = 25 to 36), where participants identified a correct word among four similar nonwords (MWT-B; Lehrl, 2005) (see Author Note 1).

A confirmatory factor analysis showed good fit for a model with four correlated ability factors (comparative fit index = 0.92, root mean square error of approximation = 0.074, standardized root mean square residual = 0.054). For the constructs that included multiple measures (i.e., reasoning, episodic memory, and speed), the scores were calculated as the average *z*-score across all measures. For all the constructs, higher scores represent higher abilities.

### Well-being

Well-being was assessed by referring to the tripartite model of subjective well-being, including positive and negative affect and life satisfaction (Cooke et al., 2016; Diener et al., 2018). *Positive affect and negative affect* were assessed with the German version of the Positive And Negative Affect Schedule (Breyer & Bluemke, 2016; Watson et al., 1988). Participants answered the question “how often you have felt this feeling during the last year” on a scale from 1 (very slightly or not at all) to 5 (extremely). Positive affect was the average score across 10 items (e.g., “excited,” “inspired”) and negative affect was the average score across 10 items (e.g., “upset,” “nervous”). Higher scores indicated higher levels of positive affect (*M* = 3.86, *SD* = 0.48, range = 2.40 to 5.00, Cronbach’s alpha = 0.79) and negative affect (*M* = 1.55, *SD* = 0.42, range = 1.00 to 2.70, Cronbach’s alpha = 0.77), respectively.

*Life satisfaction* was assessed with the German version of the Satisfaction With Life Scale (Diener et al., 1985; Janke & Glöckner-Rist, 2014). Participants indicated the extent to which they agreed or disagreed with five statements (e.g., “So far I have gotten the important things I want in life”) on a 7-point scale (1 = strongly disagree and 7 = strongly agree). Higher scores indicated higher life satisfaction (*M* = 5.41, *SD* = 1.15, range = 1.60 to 7.00, Cronbach’s alpha = 0.91).

### Covariates

We controlled for participants’ total frequency of social interactions over the study period and living status, given that fewer social interactions could contribute to higher routineness. *Total frequency of social interactions* was the total number of social interactions reported by the participants during the entire experience sampling period (*M* = 94.6, *SD* = 49.6). *Living status* was a binary variable indicating living alone (= 1) versus with other(s) (= 0). We also controlled for demographic information of age, sex, education, and health conditions. *Age* was indicated by the number of years since an individual’s birth. *Sex* was a binary variable (men = 1, women = 0). *Education* was a binary variable indicating whether a participant had a degree from a university or college of applied science (yes = 1, no = 0). *Health conditions* were measured by the number of a total 23 physician-diagnosed health conditions (e.g., diabetes, high/low blood pressure) during the last 2 years (refer to Supplementary Materials for the complete list of health conditions). Marital status was highly related to living status (*r* = 0.80) and was thus not included in our analyses.

### Analytical Approach

#### Recurrence quantification analysis

To quantify routineness, we used the method of recurrence quantification analysis (D’Mello & Gruber, 2021; Webber & Zbilut, 2005). The method analyzes time series that represent a dynamic system by identifying recurrent patterns over time. In our case, it quantified the degree to which a person maintained and returned to social interactions with certain characteristics over time. We represented social interactions of a person as a time series, where each element of the time series was a social interaction that happened at a certain time point. We then compared the time series to itself at different time delays. Overlapping points were marked as “recurrent.” Figure 1, panel B, illustrates the concept of recurrent elements of a time series used in this approach. Generally, nearby points that are a certain distance away (radius) could also be considered recurrent. This radius is defined by the lag with which the time series is compared to itself. In our study, however, we took a lag of 0 and looked for exact matches over time as the time unit was already 1 hr. The routineness score was calculated as the recurrence rate or the proportion of recurrent points in the time series. Table 1 illustrates the method with an example procedure to derive the scores of routineness of modality. The score has a possible range of 0 to 1. A score of 0 indicates no repetition and a score of 1 indicates occurring repeatedly all the time.

**Table 1.** Example Procedures to Calculate Routineness of Modality Across Seven Days

[Time, Modality] _Day_	[9, 1]_D1_	[9, 3]_D2_	[11, 5]_D3_	[9, 1]_D4_	[17, 2]_D5_	[11, 5]_D6_	[9, 1]_D7_	Sum	Score
[9, 1]_D1_	—	0	0	1	0	0	1		
[9, 3]_D2_	0	—	0	0	0	0	0		
[11, 5]_D3_	0	0	—	0	0	1	0		
[9, 1]_D4_	1	0	0	—	0	0	1		
[17, 2]_D5_	0	0	0	0	—	0	0		
[11, 5]_D6_	0	0	1	0	0	—	0		
[9, 1]_D7_	1	0	0	1	0	0	—		
#Matches	2	0	1	2	0	1	2	8	
#Max. possible matches	6	6	6	6	6	6	6	42	
#Matches/#Max. possible matches									0.19

*Note:* Routineness of modality was calculated based on interaction time and interaction modality. The tuples [Time, Modality] show all social interactions in different modality at different times of day that occurred within a person for more than 7 days. Interaction time [Time,] was in the format 0 to 24. Interaction modality [, Modality] included 1 to 7, indicating (1) face-to-face, (2) telephone, (3) e-mail, (4) text message, (5) video chat, (6) letter, and (7) social media. We marked the cell with 1 to indicate a repetition of the tuples [Time, Modality]. Otherwise, we marked it with 0. We then summed up all the matched cells (#Matches; 8) and divided them by the maximum number of possible matches (#Max. possible matches; 42), which returned the score of the routineness of modality (0.19).

We calculated four routineness scores. *Routineness of interaction* indicated the occurrence of any social interaction during the same hour of day (*M* = 0.08, *SD* = 0.01, range = 0.05 to 0.12). *Routineness of modality* was calculated based on interaction time and interaction modality, indicating social interactions using the same modality occurred during the same hour of day (*M* = 0.04, *SD* = 0.01, range = 0.01 to 0.08). In the same vein, *routineness of partner type* was calculated based on interaction time and interaction partner type (*M* = 0.05, *SD* = 0.01, range = 0.03 to 0.10). *Routineness of location* was calculated based on interaction time and interaction location (*M* = 0.05, *SD* = 0.01, range = 0.03 to 0.08).

#### Linear regression

We used linear regressions to examine relations between (1) cognitive abilities and routineness and (2) routineness and well-being. We took into account the covariates in all our analyses. The cognitive ability and well-being scores were transformed into *z*-scores (*M* = 0; *SD *= 1), and all covariates were centered at the sample average. We used the Anderson–Darling Test and the Shapiro–Wilk Test to examine normality of the residuals of the regression models, ensuring the assumption of homoscedasticity (Razali & Wah, 2011). Given that not all the residuals were normally distributed, we conducted robust regression analyses (i.e., a weighted-least-squares regression; Fox, 2019). Analyses were conducted in the R package “MASS” version 7.3-54 (Ripley et al., 2013) in R version 4.2.1 (R Core Team, 2013). We rejected the null hypothesis if the absolute *t* value was greater than 1.96, which is equivalent to *p* < .05. *R*-squared was calculated to indicate the proportion of the variance for a dependent variable that is explained by the independent variables in a regression model.

## Results

The 103 participants were aged 65 to 84 years old (*M* = 71.4, *SD *= 4.4) and had, on average, 2.09 (*SD* = 1.66) health conditions. About 60% were men, 23% had a university degree, and 39% lived alone. Bivariate correlations of key variables are displayed in Supplementary Table 2.

### Cognitive Abilities and Routineness

As shown in Supplementary Materials, routineness of social interaction in general (Supplementary Table 2), routineness of social interactions with the same modality (Supplementary Table 3), routineness of social interactions with the same partner types (Supplementary Table 4), and routineness of social interactions with the same location (Supplementary Table 5) were not significantly related to any cognitive ability, including reasoning, episodic memory, speed, or vocabulary.

### Routineness and Well-Being

**Figure 2.**
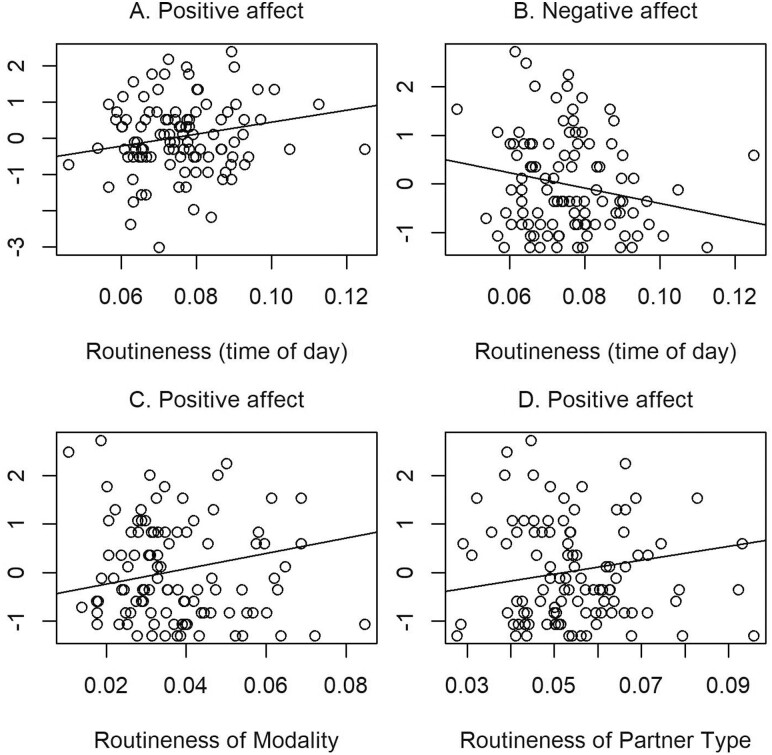
Plots for significant associations between routineness of social interactions with well-being. The dots indicate scores of participants. The lines indicate the regression estimates (Tables 2 and 3).

As shown in Table 2, higher routineness of social interaction in general (Model 1, estimate = 16.48, *SE* = 7.34, *t*(95)* *= 2.24), higher routineness of social interactions with the same modality (Model 2, estimate = 15.87, *SE* = 7.43, *t*(95)* *= 2.14) and higher routineness of social interactions with the same partner type (Model 3, estimate = 14.22, *SE* = 7.16, *t*(102)* *= 1.99) were associated with higher positive affect. As shown in Table 3, higher routineness of any social interactions was associated with lower negative affect (Model 5, estimate = −15.74, *SE* = 8.00, *t*(95)* *= −1.97). Additionally, routineness scores were not related to life satisfaction (Supplementary Table 6). Figure 2 displays the key findings.

**Table 2.** Associations Between Routineness and Positive Affect

Predictors	Model 1		Model 2		Model 3		Model 4	
	Estimate	*SE*	Estimate	*SE*	Estimate	*SE*	Estimate	*SE*
Intercept	0.07	0.09	0.07	0.09	0.06	0.09	0.06	0.10
Routineness (time)	**16.48**	7.34						
Routineness of modality			**15.87**	7.43				
Routineness of partner type					**14.22**	7.16		
Routineness of location							12.38	8.58
Total frequency	**0.01**	0.002	**0.01**	0.002	**0.01**	0.002	**0.01**	0.002
Living status (alone)	−0.08	0.22	0.001	0.23	0.004	0.23	−0.06	0.24
Age	−0.006	0.02	−0.02	0.02	−0.01	0.02	−0.003	0.02
Sex (men)	−0.30	0.22	−0.30	0.22	−0.17	0.22	−0.21	0.23
University degree (yes)	0.24	0.23	0.11	0.24	0.17	0.24	0.17	0.25
Health conditions	**−0.18**	0.06	**−0.17**	0.06	−0.17	0.06	**−0.16**	0.06
Total *R*-squared	21%		21%		19%		19%	
Δ*R*-squared	4%		4%		2%		2%	

*Note: SE* = standard error; *R*-squared = 1 − residual sum of squares/total sum of squares; Δ*R*-squared = increase in *R*-squared compared to models without the routineness predictor. Bold scores indicate significant result with absolute *t* value > 1.96.

**Table 3.** Associations Between Routineness and Negative Affect

Predictors	Model 1		Model 2		Model 3		Model 4	
	Estimate	*SE*	Estimate	*SE*	Estimate	*SE*	Estimate	*SE*
Intercept	−0.06	0.10	−0.05	0.10	−0.04	0.10	−0.03	0.10
Routineness (time)	**−15.74**	8.00						
Routineness of modality			−11.87	7.82				
Routineness of partner type					−5.25	7.67		
Routineness of location							−0.56	8.74
Total frequency	−0.003	0.002	−0.003	0.002	−0.002	0.002	−0.002	0.002
Living status (alone)	−0.08	0.24	−0.16	0.24	−0.15	0.25	−0.12	0.24
Age	−0.03	0.02	−0.02	0.03	−0.03	0.03	−0.03	0.03
Sex (men)	0.38	0.24	0.34	0.24	0.27	0.24	0.28	0.24
University degree (yes)	−0.04	0.25	0.06	0.25	0.04	0.26	0.05	0.25
Health conditions	**0.20**	0.06	**0.18**	0.06	**0.19**	0.06	**0.19**	0.06
Total *R*-squared	17%		15%		13%		13%	
Δ*R*-squared	4%		2%		0%		0%	

*Note*: *SE* = standard error; *R*-squared = 1 − residual sum of squares/total sum of squares; Δ*R*-squared = increase in *R*-squared compared to models without the routineness predictor. Bold scores indicate significant result with absolute *t* value > 1.96.

## Discussion

This study examined associations of routineness of social interactions with cognitive abilities and well-being in community-dwelling older adults. First, we examined associations between cognitive abilities and routineness of social interactions. We proposed that older adults with lower cognitive abilities would show a more routinized pattern of social interactions. We did not find any support for this hypothesis. Preference for routines that was shown to be associated with cognitive abilities in prior research (Bergua et al., 2006; Tournier et al., 2012) referred to the psychological trait of disliking disruption and liking order and routine (Reich & Zautra, 1991). The subjective report of preference for routineness may be more sensitive to cognitive decline than actual behaviors of developing and maintaining routines, although, notably, preference for routines was positively related to the likelihood of engagement in routine activities (Bouisson & Swendsen, 2003). Moreover, compared to the core activities in older adults’ daily life (e.g., waking up, breakfast), maintaining routine social interactions could be more dependent on prospective memory (which was not assessed in this study) and external cues (e.g., friends’ reminder; Altgassen et al., 2010). This may increase the complexity of the associations expected or mask them. Additionally, the nonsignificant results might be due to our sample of older adults living independently in the community with probably little decline in cognitive abilities. Although our study found no associations between cognitive abilities and routineness of social interactions, such associations may exist in more vulnerable populations.

Next, we examined associations between routineness of social interactions and well-being. Results showed that routineness of social interactions was associated with higher well-being, particularly in terms of higher positive affect. Our findings suggested that routineness of social interactions in general, of social interactions through the same modality, and of social interactions with the same type of people were associated with higher well-being. These findings are in line with the research that showed routines can promote sense of structure and meaning in life (Heintzelman & King, 2019; Mohideen & Heintzelman, 2023). The findings are also in line with the social zeitgeber theory in that routines might offer a sense of life stability (Grandin et al., 2006). In addition to the recurring time pattern that might yield feelings of structure and stability, social interactions that took place regularly may promote older adults' well-being through fulfilling the need to belong while consuming relatively little energy (Hall et al., 2021). Our findings are different from the research that proposed routineness could lead to lower well-being, due to the reduction of complexity and the increase of inflexibility in life (Bouisson & Swendsen, 2003). Similar to our reasoning above, our sample of independent and active community-dwelling older adults might have a high level of autonomy to flexibly coordinate their social life. Thus, routineness may be pleasant agentic choices rather than unpleasant compromise. Further, it is important to note that the ranges of the routineness scores were up to 0.12. That is, only up to 12% social interactions out of all observations were routine. Routineness of social interactions might have a different association with well-being, when it surpasses a certain threshold becoming overly repetitive and insufficiently stimulating. Additional evidence is needed for further investigating routineness in relation to well-being. 

Notably, routineness of social interactions was not related to life satisfaction. Our findings suggest that routineness of social interactions was more related to the affective experience than the cognitive evaluation of well-being, according to the tripartite model of subjective well-being (Diener et al., 2018). This seems to be reasonable, as affective well-being could be more reactive toward specific events and activities than cognitive well-being, which is evaluated referencing to global life circumstances (Luhmann et al., 2012). Additionally, positive affect (range = 2.40 to 5.00) had more variance than negative affect (range = 1.00 to 2.70) in our sample, and this may explain the presence of more significant associations with positive affect than with negative affect. In sum, routineness had a positive association with affective well-being in our sample of community-dwelling older adults who could organize their social life autonomously.

This study was innovative in taking a combination of the event-contingent experience sampling design and the recurrence quantification analysis to quantify routineness of social interactions of older adults. Yet, there are several limitations to be considered. First, we could not address temporal directionality of associations between routineness and cognition/well-being in our single-wave experience sampling design. For example, happiness could lead to higher satisfaction with relationships over time (Lyubomirsky et al., 2005). Thus, higher positive affect might encourage older adults to establish and maintain routine social meetings. Relatedly, we focused on between-person trait-level associations between routineness and cognitive abilities and well-being, but the associations could be different at the state level within individuals. For example, higher routineness may associate with lower well-being, at moments when one feels bored and desires novelty (Bench & Lench, 2013). These complexities warrant future investigation.

Second, we conceptualized social interactions as a result of an agentic decision, but they could also be determined by external schedules, such as church meetings, although individuals could still choose not to take part in these regular meetings. Future research could take into account more contextual characteristics to further quantify routineness of social interactions, such as whether the interaction was “voluntary” versus “involuntary” (Hall et al., 2021) and the duration of social interactions (e.g., a brief 6-min exchange versus a 2-hr meeting).

Third, referring to prior research (Bouisson & Swendsen, 2003; Monk et al., 1991), we operationalized routineness as events that reoccurred at the same hour of day. However, social interactions that happened at neighboring hours, such as 1:50 pm and 2:20 pm, could be classified as routine interactions, but they were not indicated as routine in our operational method. Relatedly, routineness could also be events that reoccurred on the same day of the week or of the year (Tighe et al., 2015). Arguably, routineness quantified by time of day may be closer related to the stability of biological circadian rhythms and thus well-being, according to the social zeitgeber theory (Grandin et al., 2006). Moreover, our 3-week study was too short to examine routineness spanning over weeks or years. Future research could consider examining routineness in a different timescale in relation to older adults’ health and well-being.

Finally, our study included a sample of active community-dwelling older adults from Switzerland. Research needs to examine whether the findings generalize to other older populations (e.g., clinical samples). To gain a comprehensive view on routineness in older adults’ daily life, future studies could examine routineness of other activity domains and utilize different naturalistic observation methods (Röcke et al., 2023).

## Supplementary Material

gbae057_suppl_Supplementary_Tables_S1-S6

## Data Availability

The study was not preregistered. Data and detailed analyses code and output are shared on OSF (https://osf.io/ekfzc/).

## Author Note

1. We did not have the data at the item level to calculate the Cronbach’s alpha for the tests of episodic memory and vocabulary. Notably, the test of vocabulary is widely used in research in German-speaking countries and thus could be seen as a reliable measure (Lehrl et al., 1995). The episodic memory measures are highly similar to widely used measures in large-scale longitudinal studies with older adults.
