# Supplementary material for: Routineness of Social Interactions Is Associated With Higher Affective Well-Being in Older Adults
Source: J Gerontol B Psychol Sci Soc Sci. 2024 Apr 10;79(6):gbae057. doi: 10.1093/geronb/gbae057 (PMC11075731; doi:10.1093/geronb/gbae057)
Supplement: gbae057_suppl_Supplementary_Tables_S1-S6 [file gbae057_suppl_supplementary_tables_s1-s6.docx]

***The Journals of Gerontology, Series B: Psychological Sciences and Social Sciences* Supplementary Material: Supplementary Material: Minxia Luo, Kristina Yordanova, Birthe Macdonald, & Gizem Hülür. Routineness of Social Interactions is Associated with Higher Affective Well-Being in Older Adults.**

**Question on Health Conditions**

Have you suffered from the following diseases / symptoms in the past 2 years or have you been treated for them?

1. Asthma, bronchitis, emphysema of old age
2. Tuberculosis
3. Lung complaints
4. Arthritis, rheumatism or other diseases of the bone apparatus
5. Lumbago, recurrent back pain
6. Persistent (persistent) skin problems (e.g. eczema)
7. Thyroid gland diseases
8. High fever
9. Recurring stomach problems, diarrhea
10. Gall bladder problems
11. Problems with blood vessels
12. AIDS, HIV infection
13. Lupus or other autoimmune diseases
14. Toothache
15. High / low blood pressure
16. Anxiety, depression, or other emotional illness
17. Alcohol or drug problems
18. Migraine
19. Chronic sleep problems
20. Diabetes
21. Multiple sclerosis, epilepsy or other neurological diseases
22. Stroke
23. Cancer

Table S1. Bivariate Correlations Among Key Variables*.*

| Variable | 1 | 2 | 3 | 4 | 5 | 6 | 7 | 8 | 9 | 10 | 11 | 12 | 13 | 14 | 15 |
| --- | --- | --- | --- | --- | --- | --- | --- | --- | --- | --- | --- | --- | --- | --- | --- |
| 1. Routineness (time) |  |  |  |  |  |  |  |  |  |  |  |  |  |  |  |
| 2. Routineness of modality | **.43** |  |  |  |  |  |  |  |  |  |  |  |  |  |  |
| 3. Routineness of partner type | **.57** | **.33** |  |  |  |  |  |  |  |  |  |  |  |  |  |
| 4. Routineness of location | **.59** | **.24** | **.53** |  |  |  |  |  |  |  |  |  |  |  |  |
| 5. Reasoning | -.11 | -.14 | -.06 | .02 |  |  |  |  |  |  |  |  |  |  |  |
| 6. Memory | .07 | **-.24** | .03 | .04 | **.38** |  |  |  |  |  |  |  |  |  |  |
| 7. Speed | -.02 | -.09 | .04 | .07 | **.53** | **.31** |  |  |  |  |  |  |  |  |  |
| 8. Vocabulary | .08 | **.22** | .13 | .01 | **.33** | **.19** | **.37** |  |  |  |  |  |  |  |  |
| 9. Positive affect | .08 | .10 | .16 | .11 | .12 | .15 | .04 | .11 |  |  |  |  |  |  |  |
| 10. Negative affect | -.14 | -.12 | -.07 | -.02 | .01 | -.08 | -.15 | -.17 | **-.26** |  |  |  |  |  |  |
| 11. Total frequency | **-.22** | **-.32** | .01 | -.11 | **.28** | .15 | .18 | .05 | **.29** | -.04 |  |  |  |  |  |
| 12. Living status (alone) | .00 | -.08 | -.13 | -.01 | -.13 | .00 | .09 | .17 | -.06 | -.11 | -.14 |  |  |  |  |
| 13. Age | .08 | **.30** | .09 | .03 | -.16 | **-.31** | -.15 | .18 | -.14 | -.06 | **-.27** | **.32** |  |  |  |
| 14. Sex (men) | .13 | **.24** | .02 | .05 | **.19** | -.09 | -.09 | .05 | -.04 | .15 | -.01 | **-.46** | -.04 |  |  |
| 15. University degree (yes) | -.04 | .16 | .05 | .06 | **.43** | .12 | .18 | **.29** | .06 | .02 | .00 | -.16 | .04 | **.31** |  |
| 16. Health conditions | .07 | -.10 | .02 | -.04 | -.16 | -.05 | -.10 | -.02 | **-.28** | **.28** | -.03 | .10 | .15 | -.16 | -.16 |

*Note*. Bolded scores are *p*-value < .05.

Table S2. *Associations Between Cognitive Abilities and Routineness of Social Interaction in General*

| Predictors | Model 1 | | Model 2 | | Model 3 | | Model 4 | |
| --- | --- | --- | --- | --- | --- | --- | --- | --- |
|  | Estimate | SE | Estimate | SE | Estimate | SE | Estimate | SE |
| Intercept | **0.08** | 0.001 | **0.08** | 0.001 | **0.08** | 0.001 | **0.08** | 0.001 |
| Reasoning | 0.000 | 0.001 |  |  |  |  |  |  |
| Episodic memory |  |  | 0.001 | 0.001 |  |  |  |  |
| Speed |  |  |  |  | 0.002 | 0.001 |  |  |
| Vocabulary |  |  |  |  |  |  | 0.001 | 0.001 |
| Total frequency | 0.000 | <0.001 | 0.000 | <0.001 | **0.000** | <0.001 | 0.000 | <0.001 |
| Living status (alone) | 0.001 | 0.003 | 0.001 | 0.003 | 0.001 | 0.003 | 0.001 | 0.003 |
| Age | 0.000 | <0.001 | 0.000 | <0.001 | 0.000 | <0.001 | 0.000 | <0.001 |
| Sex (men) | 0.004 | 0.003 | 0.01 | 0.003 | 0.01 | 0.003 | 0.004 | 0.003 |
| University degree (yes) | -0.003 | 0.003 | -0.004 | 0.003 | -0.005 | 0.003 | -0.004 | 0.003 |
| Health conditions | 0.001 | 0.001 | 0.001 | 0.001 | 0.001 | 0.001 | 0.001 | 0.001 |

*Note*. Est. = estimate; SE = standard error. Bold scores indicate significant result with absolute t value > 1.96*.*

Table S3. *Associations Between Cognitive Abilities and Routineness of Social Interactions with the Same Modality*

| Predictors | Model 1 | | Model 2 | | Model 3 | | Model 4 | |
| --- | --- | --- | --- | --- | --- | --- | --- | --- |
|  | Estimate | SE | Estimate | SE | Estimate | SE | Estimate | SE |
| Intercept | **0.04** | 0.001 | **0.04** | 0.001 | **0.04** | 0.001 | **0.04** | 0.001 |
| Reasoning | -0.003 | 0.001 |  |  |  |  |  |  |
| Episodic memory |  |  | -0.002 | 0.001 |  |  |  |  |
| Speed |  |  |  |  | -0.001 | 0.001 |  |  |
| Vocabulary |  |  |  |  |  |  | 0.002 | 0.001 |
| Total frequency | **0.000** | <0.001 | **0.000** | <0.001 | **0.000** | <0.001 | **0.000** | <0.001 |
| Living status (alone) | -0.004 | 0.003 | -0.004 | 0.003 | -0.004 | 0.003 | -0.005 | 0.003 |
| Age | **0.001** | <0.001 | **0.001** | <0.001 | **0.001** | <0.001 | **0.001** | <0.001 |
| Sex (men) | 0.004 | 0.003 | 0.003 | 0.003 | 0.003 | 0.003 | 0.003 | 0.003 |
| University degree (yes) | **0.007** | 0.004 | 0.005 | 0.003 | 0.005 | 0.003 | 0.003 | 0.004 |
| Health conditions | -0.001 | 0.001 | -0.001 | 0.001 | -0.001 | 0.001 | -0.001 | 0.001 |

*Note*. Est. = estimate; SE = standard error. Bold scores indicate significant result with absolute t value > 1.96*.*

Table S4. *Associations Between Cognitive Abilities and Routineness of Social Interactions with the Same Partner Type*

| Predictors | Model 1 | | Model 2 | | Model 3 | | Model 4 | |
| --- | --- | --- | --- | --- | --- | --- | --- | --- |
|  | Estimate | SE | Estimate | SE | Estimate | SE | Estimate | SE |
| Intercept | **0.05** | 0.001 | **0.05** | 0.001 | **0.05** | 0.001 | **0.05** | 0.001 |
| Reasoning | -0.002 | 0.001 |  |  |  |  |  |  |
| Episodic memory |  |  | 0.000 | 0.001 |  |  |  |  |
| Speed |  |  |  |  | 0.001 | 0.001 |  |  |
| Vocabulary |  |  |  |  |  |  | 0.001 | 0.001 |
| Total frequency | 0.000 | <0.001 | 0.000 | <0.001 | 0.000 | <0.001 | 0.000 | <0.001 |
| Living status (alone) | -0.004 | 0.003 | -0.004 | 0.003 | -0.004 | 0.003 | -0.004 | 0.003 |
| Age | 0.000 | <0.001 | 0.000 | <0.001 | 0.000 | <0.001 | 0.000 | <0.001 |
| Sex (men) | -0.003 | 0.003 | -0.003 | 0.003 | -0.003 | 0.003 | -0.003 | 0.003 |
| University degree (yes) | 0.005 | 0.003 | 0.003 | 0.003 | 0.003 | 0.003 | 0.002 | 0.003 |
| Health conditions | 0.000 | 0.001 | 0.000 | 0.001 | 0.000 | 0.001 | 0.000 | 0.001 |

*Note*. SE = standard error. Bold scores indicate significant result with absolute t value > 1.96*.*

Table S5. *Associations Between Cognitive Abilities and Routineness of Social Interactions with the Same Location*

| Predictors | Model 1 | | Model 2 | | Model 3 | | Model 4 | |
| --- | --- | --- | --- | --- | --- | --- | --- | --- |
|  | Estimate | SE | Estimate | SE | Estimate | SE | Estimate | SE |
| Intercept | **0.05** | 0.001 | **0.05** | 0.001 | **0.05** | 0.001 | **0.05** | 0.001 |
| Reasoning | 0.001 | 0.001 |  |  |  |  |  |  |
| Episodic memory |  |  | 0.001 | 0.001 |  |  |  |  |
| Speed |  |  |  |  | 0.001 | 0.001 |  |  |
| Vocabulary |  |  |  |  |  |  | 0.001 | 0.001 |
| Total frequency | 0.000 | <0.001 | 0.000 | <0.001 | 0.000 | <0.001 | 0.000 | <0.001 |
| Living status (alone) | -0.001 | 0.003 | -0.001 | 0.003 | -0.001 | 0.003 | -0.001 | 0.003 |
| Age | 0.000 | <0.001 | 0.000 | <0.001 | 0.000 | <0.001 | 0.000 | <0.001 |
| Sex (men) | -0.001 | 0.003 | -0.001 | 0.003 | -0.001 | 0.003 | -0.001 | 0.003 |
| University degree (yes) | 0.002 | 0.003 | 0.002 | 0.003 | 0.002 | 0.003 | 0.003 | 0.003 |
| Health conditions | 0.000 | 0.001 | 0.000 | 0.001 | 0.000 | 0.001 | 0.000 | 0.001 |

*Note*. SE = standard error. Bold scores indicate significant result with absolute t value > 1.96*.*

Table S6. *Associations Between Routineness of Social Interactions and Life Satisfaction*

| Predictors | Model 1 | | Model 2 | | Model 3 | | Model 4 | |
| --- | --- | --- | --- | --- | --- | --- | --- | --- |
|  | Estimate | SE | Estimate | SE | Estimate | SE | Estimate | SE |
| Intercept | 0.06 | 0.10 | 0.06 | 0.09 | 0.05 | 0.10 | 0.05 | 0.10 |
| Routineness (time) | 9.65 | 7.72 |  |  |  |  |  |  |
| Routineness of modality |  |  | 12.65 | 7.15 |  |  |  |  |
| Routineness of partner type |  |  |  |  | 0.15 | 7.32 |  |  |
| Routineness of location |  |  |  |  |  |  | 2.08 | 8.40 |
| Total frequency | **0.01** | 0.002 | **0.01** | 0.002 | **0.004** | 0.002 | **0.004** | 0.002 |
| Living status (alone) | -0.13 | 0.23 | -0.06 | 0.22 | 0.11 | 0.24 | -0.12 | 0.23 |
| Age | -0.01 | 0.02 | -0.02 | 0.02 | -0.01 | 0.02 | -0.01 | 0.02 |
| Sex (men) | -0.10 | 0.22 | -0.10 | 0.22 | -0.04 | 0.23 | -0.04 | 0.23 |
| University degree (yes) | -0.33 | 0.24 | -0.42 | 0.23 | -0.38 | 0.24 | -0.38 | 0.24 |
| Health conditions | **-0.19** | 0.06 | **-0.18** | 0.06 | **-0.19** | 0.06 | **-0.19** | 0.06 |

*Note*. Est. = estimate; SE = standard error. Bold scores indicate significant result with absolute t value > 1.96*.*
